# Dialysis Adequacy: A Cross-Sectional Study to Assess the Reliability of the Online Clearance Monitor to Measure Dialysis Dose

**DOI:** 10.7759/cureus.52328

**Published:** 2024-01-15

**Authors:** Arul Rajagopalan, Niranjan Raja, Gandhi Mohan

**Affiliations:** 1 Department of Nephrology, Coimbatore Medical College and Hospital, Coimbatore, IND; 2 Department of Nephrology, Mahatma Gandhi Medical College and Research Institute, Puducherry, IND

**Keywords:** kt/v, hemodialysis, online clearance monitor, urea reduction ratio, dialysis dose, chronic kidney disease (ckd)

## Abstract

Background

Frequent assessment of the dialysis dose delivered to hemodialysis patients might help improve morbidity and mortality. Daugirdas' second-generation formula is the recommended method for calculating dialysis doses. However, urea reduction ratios (URRs) and online clearance monitors (OCMs) are frequently used to assess dialysis adequacy due to their more straightforward concept and ease of use. This study was conducted to determine the most reliable method for measuring dialysis adequacy by comparing the correlation of urea reduction ratio and online clearance monitor measurements with the dialysis dose measured using the recommended Daugirdas' second-generation formula.

Methods

This study was an observational, cross-sectional, single-center study. The dialysis dose was measured as a urea reduction ratio and by an online clearance monitor simultaneously for 50 patients. It was compared to the dialysis dose measurements obtained using Daugirdas' second-generation formula.

Results

There was a statistically significant strong positive correlation (r = 0.929; p ≤ 0.001) of the urea reduction ratio and a poor concordance (ρC = 0.401; p ≤ 0.001) of online clearance monitor measurements with the dialysis dose measured using Daugirdas' second generation formula.

Conclusion

Our findings illustrate that the urea reduction ratio may be a more straightforward and reliable means for assessing the adequacy of intermittent hemodialysis with minimal errors in patients compared to online clearance monitors. Online clearance monitors offer easy estimation and practicality with minimal effort but are prone to multiple errors and may not be accurate in some settings.

## Introduction

Chronic kidney disease (CKD) is one of the leading causes of mortality and morbidity worldwide, and its global prevalence has increased by nearly one-third since the 1990s [1].

Kidney disease improving global outcomes (KDIGO) divides CKD into five categories based on estimated glomerular filtration rate (eGFR) [2], of which the last category of patients, category G5, previously known as end-stage kidney disease (ESRD), usually requires some form of renal replacement therapy (RRT) for the sustenance of life. Hemodialysis (HD) is one of the most common modes of RRT in which a solute passively diffuses down its concentration gradient from one fluid compartment (either blood or dialysate) into the other [3]. The goal of hemodialysis is the removal of toxins from the body to maintain homeostasis. The optimal dialysis dose significantly reduces morbidity and mortality in these patients, as shown in numerous outcome studies [4-6]. Hence, knowing the dialysis dose delivered helps improve health in hemodialysis, making it an essential quality parameter of chronic HD therapy.

The fundamental measure of dialysis adequacy is the comparison of concentrations of a defined substance in the blood before and after a dialysis session. Urea is used to measure dialysis adequacy as it is considered a surrogate of other uremic toxins and is dialyzed easily. It is a small solute, making urea Kt/V a sensitive measure of the overall dialysis dose [7]. Treatment-related urea reduction ratio (URR) and the kinetic model of urea (UKM) Kt/V are used to measure dialysis adequacy. The standard method for determining dialysis dose includes three steps: pre- and post-dialysis blood samples, analysis of the urea concentration in the collected samples, and finally, application of urea kinetic modeling or, more commonly, the Daugirdas second-generation formula [8].

A more straightforward method, direct measurement of dialysis adequacy, URR, may be used as a substitute since the relative decrease in urea concentration during therapeutic dialysis is the most significant determinant of Kt/V. URR fails to include both the urea generation and the contraction in extracellular volume that occurs during routine HD, resulting in variability even within individual patients. Nonetheless, when outcomes are compared with either URR or Kt/V, there is no/minimal detectable difference in the degree of correlation [4,5].

A still simpler method to probe dialysis adequacy is by using an online clearance monitor (OCM). This method does not require blood draws and can predict the delivered Kt/V in real time. It is based on the assumption that changes in dialysate conductivity are caused by the transmembrane movement of small electrolytes, primarily sodium. Sodium ions are the major ions in the dialysis solution, and their concentration determines the dialysis fluid's total conductivity. Changes in dialysate conductivity can be measured by the sensors installed in the inflow and outflow lines of the dialyzer within the newer dialysis machines. Both urea and sodium exhibit comparable diffusion characteristics across a synthetic dialysis membrane, i.e., their specific diffusion coefficient is almost identical at 37 °C (Na+: 1.94 × 10^−5^ cm^2^/s; urea: 2.20 × 10^−5^ cm^2^/s). Under actual dialysis conditions, the difference in clearance is smaller than in diffusion coefficients because clearance is limited by blood and dialysate flow rates and not by the diffusion characteristics across the dialyzer membrane.

The hemodialysis machine generates short-term pulses of sodium, increasing its concentration in the dialysis fluid. This transient increase in the conductivity of the dialysis fluid before entering the dialyzer is subsequently reduced by the diffusion of a portion of the sodium ions across the dialysis membrane into the patient's blood. As urea has similar diffusion properties to that of the sodium ion, urea clearance can be determined (using appropriate correction factors) irrespective of the actual urea concentration in the blood. Since all these steps occur automatically in the hemodialysis machine, the dialysis dose can be assessed more frequently with the least effort by measuring conductivity clearance across the dialyzer membrane.

The National Kidney Foundation's (NKF) Kidney Disease Outcomes Quality Initiative (KDOQI) and European Best Practice Guidelines for Hemodialysis recommend measuring the dialysis dose at least monthly [9,10]. This study compared the practical and easy measurements of dialysis dose by URR and Kt/V estimated with an online clearance monitor with those of Kt/V calculated using the Daugirdas formula.

## Materials and methods

This study was an observational, cross-sectional, single-center study comparing the urea reduction ratio and Kt/*V* calculated using the Daugirdas formula (Kt/*V*_Dau_) with the Kt/*V* estimated with an online clearance monitor (Kt/*V*_OCM_).

Patient selection and data collection

Patients on maintenance hemodialysis for more than three months were enrolled, while those under 18 years of age or with acute illnesses were excluded. A total of 50 patients on maintenance hemodialysis were enrolled in the study. We collected the demographic details and medical history with a proforma. Blood samples were taken during the regular monthly investigations. The pre-dialysis blood sample was drawn before injecting any potential diluents. The post-dialysis blood samples were drawn from the dialyzer inflow port using a stop-dialysate-flow method (three minutes) or a slow-flow method (reducing blood flow to 100 mL/min for 15 seconds).

Measurement of dialysis dose

The dialysis dose was assessed using the following methods simultaneously:

Calculation of Urea Reduction Ratio

URR = (U_pre_ - U_post_)/U_pre_ x 100 = (1 - U_post_/U_pre_) x 100

*U*_pre_ is the pre-dialysis blood urea nitrogen (sampled just before dialysis initiation) and *U*_post_ is the post-dialysis blood urea nitrogen (sampled immediately after the dialysis session). NKF KDOQI Clinical Practice Guidelines 2006 recommended a minimum URR of 65% and a target URR of 70% for patients dialyzed three times per week. URR depends on the clearance of urea, the volume of urea distribution in a patient, and the dialysis treatment duration. URR is commonly used due to its simpler concept and ease of use.

Calculation of Kt/V (Kt/V_Dau_)

Dialysis dose = (K_urea_ x T_d_)/V_urea_

*K*_urea_ is the urea clearance, *T*_d_ is the effective dialysis duration, and *V*_urea_ is the volume of urea distribution. Kt/*V* is a dimensionless formula. Gotch and Sargent introduced Kt/*V* during the revision of the National Cooperative Dialysis Study (NCDS) [11].

The most common model used for calculating Kt/*V* is the single-pool variable (spKt/*V*) volume. In daily practice, spKt/*V* may be computed based on the classic second-generation Daugirdas equation.

Kt/VDau = −ln (R-0.008×t) + (4-3.5×R)0.55 × UF/V

-ln is the negative natural logarithm, *R *is the post-dialysis urea/pre-dialysis urea, *t *is the dialysis time in hours, UF is the weight loss in kilograms, and *V* is the anthropometric urea distribution volume in liters.

To obtain the dialysis dose Kt/V, the urea distribution volume 'V' is required. Several anthropometric estimations are available; the Watson equation was applied in our study [12]. Anthropometric formulas for 'V' were shown to overestimate V in HD patients by approximately 15% on average [13]. However, this systematic overestimation of 'V' may protect the patient from underdialysis.

Prediction of Kt/V (Kt/V_OCM_)

The dialysis dose was estimated by the OCM using the Watson estimation for V (Kt/*V*_OCM_). The OCM automatically determines the urea clearance *K* based on several consecutive measurements (usually every 25 minutes by increasing conductivity to <13.9 mS/cm and lowering to >14.6 mS/cm with each pulse lasting eight seconds) spread over the dialysis treatment. A total of 50 dialysis sessions were measured using OCM. With the effective dialysis duration 't' given, the effective cleared plasma Kt is easily calculated. The clearance monitors used were 4008 S hemodialysis machines from Fresenius Medical Care, Germany, with the Online Clearance Monitor.

Sample Size Determination

Kuhlmann et al. [14] reported that the correlation between Kt/*V*_OCM_ and Kt/*V*_Dau_ is 0.950. Then, the minimum sample size required to conduct the study is '*n*'

$n=\frac{\left ( Z_{1-\alpha}+Z_{\beta} \right )}{C^{2}} + 3$

where C=0.5*$ln\frac{1+r}{1-r}$ and r is the correlation coefficient.

Then, n=7 at 99% confidence level and 90% power.

In addition, the correlation between URR and Kt/*V*_Dau_ was reported as 0.953 in the study by Moret et al. [15]. Therefore, a minimum of seven samples were required to conduct the study. We included 50 samples to reduce the sampling error.

Hemodialysis Parameters

Hemodialysis was carried out as four-hour H.D. sessions (however, duration was limited for some patients who developed acute complications during dialysis) three times a week with F6 HPS (Fresenius Medical Care) polysulfone dialyzers. A mean blood flow rate of 200 mL/min (180-220 mL/min) was obtained during the dialysis sessions with a dialysate flow of 500 mL/min in all machines. Dialysate fluid used was composed of 130-140 mEq/L of sodium, 2 mEq/L of potassium, 3 mEq/L of calcium, 0.75 mEq/L of magnesium, and 32 mEq/L of bicarbonate.

Statistical analyses

We used Statistical Package for the Social Sciences (SPSS) 20.0 version (IBM Corp., Armonk, NY) and R-programming (RStudio, Boston, MA) for analyzing the data. Descriptive statistics such as frequency distribution and mean (S.D.) were used to present the categorical and continuous variables, respectively. The coefficient of skewness was used to assess the normality diagnosis of the continuous data. Exponential curve fitting was used to find the exponential relationship between URR and Kt/*V*_Dau_. Lin's concordance correlation coefficient and Bland-Altman plot (difference plot) were used to assess the agreement between the two measurements.

## Results

Fifty hemodialysis dialysis patients were included in the study, of whom 38 (76%) were males and 12 (24%) were females. The average age of the patients was 54.32 ± 10.73 years, BMI was 20.12 ± 3.89 kg/m^2^, and dialysis vintage was 19.68 ± 5.96 months.

Diabetic kidney disease was the most common native kidney disease seen in 18 (36%) patients, followed by chronic glomerulonephritis in 9 (18%). Four (8%) had chronic interstitial nephritis, three (6%) had obstructive uropathy, three (6%) had hypertensive nephrosclerosis, and two (4%) had autosomal dominant polycystic kidney disease. Native kidney disease was not known in 11 (22%) patients, as it is common here for many patients to present with advanced stages of chronic kidney disease. Most of the patients had arteriovenous fistulas for dialysis access, while four (8%) were on a permanent catheter (Table 1).

**Table 1:** Demographic details and clinical characteristics of the patients.

Demographic and clinical variables	
Sex	
Male, n (%)	38 (76)
Female, n (%)	12 (24)
Age (years) mean ± SD	54.32 ± 10.73
BMI (kg/m^2^) mean ± SD	20.12 ± 3.89
Dialysis vintage (months) mean ± SD	19.68 ± 5.96
Native kidney disease	
Diabetic kidney disease, n (%)	18 (36)
Chronic glomerulonephritis, n (%)	9 (18)
Chronic interstitial nephritis, n (%)	4 (8)
Obstructive uropathy, n (%)	3 (6)
Hypertension, n (%)	3 (6)
ADPKD, n (%)	2 (4)
Unknown, n (%)	11 (22)
Dialysis access	
Arteriovenous fistula, n (%)	46 (92)
Permanent catheter, n (%)	4 (8)

Normality verification of the continuous data was verified using skewness statistics (Table 2). URR, Kt/V, and OCM had a skewness coefficient close to zero, concluding that the data were almost symmetrically distributed. Accordingly, parametric statistical tests were used to analyze the data.

**Table 2:** Normality verification and mean of the variables URR, Kt/VDau, and Kt/VOCM

Variables	Mean	Skewness	Standard error
URR	56.98 ± 16.27	0.832	0.662
Kt/*V*_Dau_	1.10 ± 0.46	0.814	0.337
Kt/*V*_OCM_	1.18 ± 0.41	0.766	0.337

**Figure 1:**
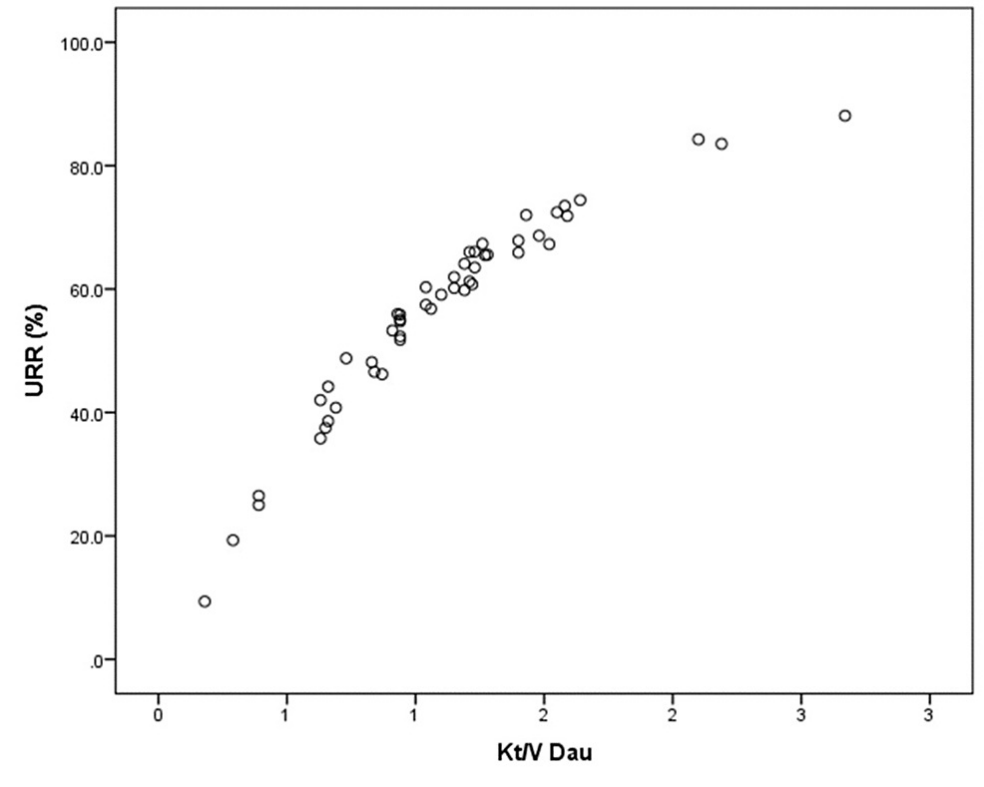
Correlation analysis between URR and Kt/VDau Showed a close curvilinear relationship between the two methods (an outlier due to measurement error was removed)

**Figure 2:**
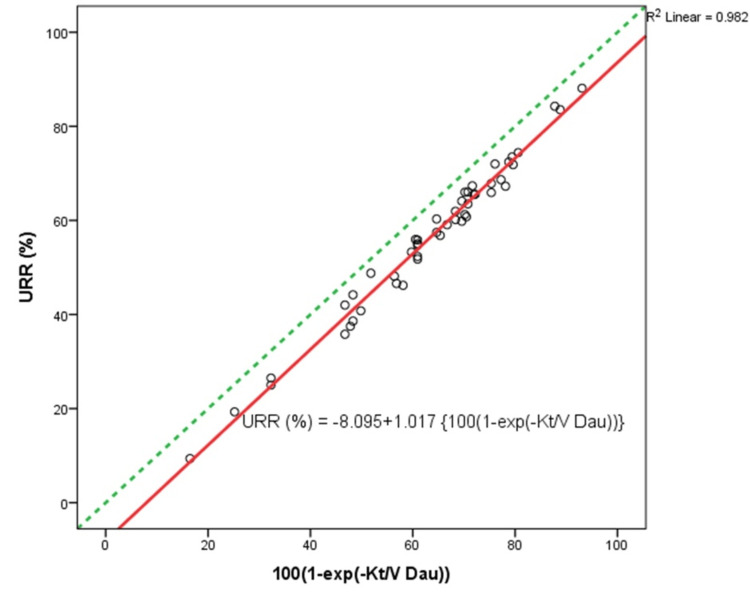
Scatter plot showing the strong linear relationship between URR and 100 {1 – exp [–Kt/VDau]} All observations lie below the identity line (green line) as URR underestimates dialysis adequacy compared to Kt/*V*_Dau_ (an outlier due to measurement error was removed)

A close curvilinear relationship is illustrated in our study by a plot of Kt/*V*_Dau_ versus URR (Figure 1). This near-perfect linear correlation between URR and 100 {1 - exp [−Kt/*V*_Dau_]} is given by R2 = 0.982 (Figure 2, Table 3). Because the URR does not account for urea generation and variable urea distribution volume reduction during dialysis, it is expected to underestimate dialysis clearance compared to Kt/*V*_Dau_, as seen by all values falling below and to the right of the identity line (Figure 2).

**Table 3:** Correlation coefficient of URR, Kt/VDau, and Kt/VOCM

		Kt/*V*_Dau_
URR	Correlation	
	R^2^	0.982
	p-value	<0.001
Kt/*V*_OCM_	Lin’s concordance	
	ρ_C_	0.401
	p-value	<0.001

If variable volume and urea generation rate are not taken into account during dialysis, single-pool, fixed-volume urea kinetics should predict an exponential decay of urea concentration. *U*_post_ = *U*_pre_ exp(−Kt/*V*_spfv_). Thus, URR = 100 (1 − *U*_post_/*U*_pre_) = 100 {1 − exp(−Kt/*V*_spfv_)} or Kt/*V*_spfv_ = −ln[1 − URR/100].

Since Kt/*V*_spfv_ should approximate Kt/*V*_Dau_, we would expect a close linear relationship between URR and 100 {1 − exp(−Kt/*V*_Dau_)}. This is illustrated in our study by a scatter plot of URR versus 100 {1 − exp(−Kt/*V*_Dau_)} (Figure 2). The linear correlation equations derived were URR (%) = −8.095 + 1.017 {100(1 − exp(−Kt/*V*_Dau_))} and Kt/*V*_Dau_ = −ln [(109.79 − URR)/101.7]. These equations will allow the calculation of Kt/*V*_Dau_ from the URR and vice versa.

**Figure 3:**
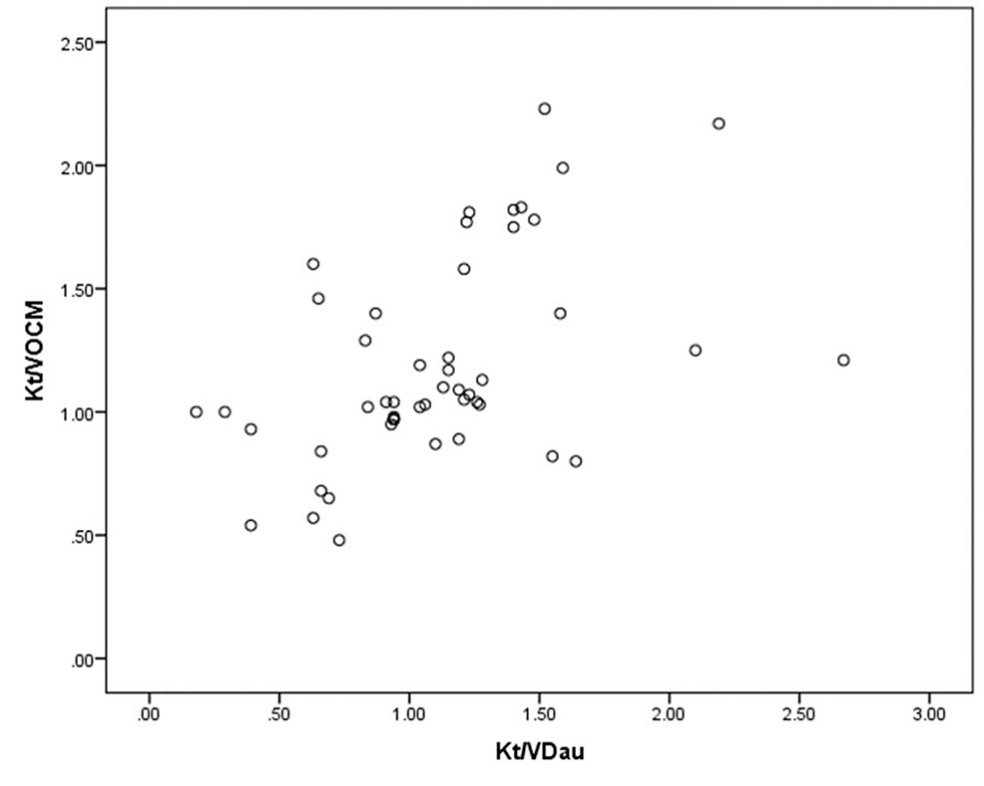
Scatter plot between Kt/VDau and Kt/VOCM A poor correlation was observed between dialysis doses measured with OCM and Daugirdas formula

**Figure 4:**
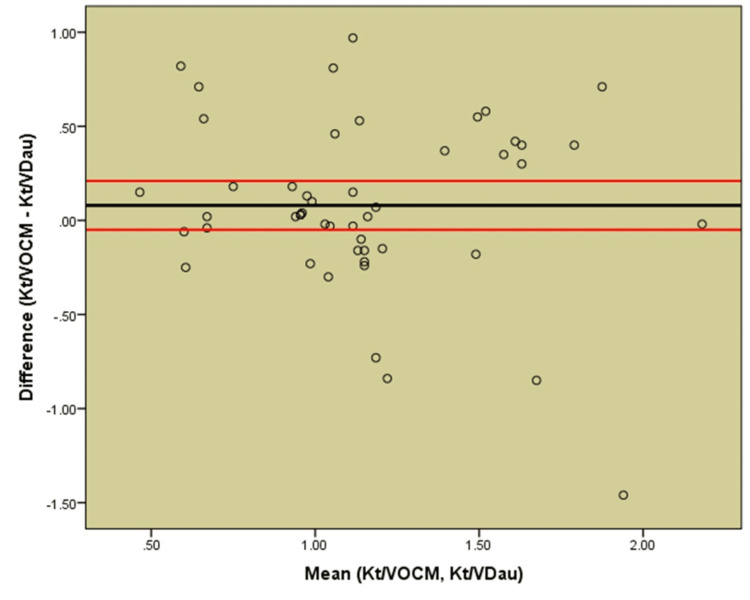
Bland Altman analysis of concordance between Kt/VOCM and Kt/VDau Bland-Altman plot for the differences between the conventional method with urea concentrations from blood samples and Daugirdas’ formula (Kt/VDau) and the automatic OCM

The direct correlation between Kt/*V*_Dau_ and Kt/*V*_OCM_ is depicted by a scatter plot showing poor correlation (Figure 3). Lin's concordance coefficient assessed the agreement between Kt/*V*_OCM_ and Kt/*V*_Dau_ (Table 3). The coefficient was 0.401 (<0.9), which indicated a poor agreement between Kt/*V*_OCM_ and Kt/*V*_Dau_, graphically represented in the Bland-Altman plot (Figure 4). On average, OCM overestimated Kt/*V* by 0.08 compared to Kt/*V*_Dau_. Most of the difference points (>50%) were scattered beyond the control limits, revealing poor agreement between the two methods.

## Discussion

Dialysis adequacy is one of the key performance indicators of a hemodialysis unit. It is preferable to have an easily measurable and reproducible index of dialysis dose that corresponds with the patient's quality of life and clinical outcomes. Three different methods of dialysis dose measurement were undertaken simultaneously and compared. Kt/*V*_Dau_ is considered the standard for routine use as its total error is very low throughout the investigated range of dialysis doses [16] and is the recommended method to measure dialysis adequacy by NKF-KDOQI [9]. However, URR and Kt/*V*_OCM_ are being widely used for a more straightforward concept and ease of measurement that may aid frequent assessments. In this study, we have compared the correlation between URR and Kt/*V*_Dau_ and, similarly, the concordance of Kt/*V*_OCM_ and Kt/*V*_Dau_.

Our study showed a poor correlation (r = 0.401) between Kt/*V*_OCM_ and Kt/*V*_Dau_. A similar poor correlation between OCM and Daugirdas's formula was established in multiple studies [17,18]. Grzegorzewska and Banachowicz made a similar comparison with 40 patients by repeating the measurements twice and obtained poor correlation r = 0.559 and 0.493 in both instances [19,20]. The probable reasons could be due to the nature of OCM measurements and the inaccurate estimation of volume 'V'. Ionic dialysance does not measure convective clearance; hence, clearance due to varying ultrafiltration in different patients was not accounted for. The best method is to collect the whole spent dialysate for clearance measurement, which would have given a better correlation [20]. Still, it is cumbersome and defeats the advantage of the ease of measuring Kt/*V* with an online clearance monitor.

Though the mean value of Kt/*V*_OCM_ was only 0.08 more than Kt/*V*_Dau_, which is within an acceptable analytical error range of ≈ ±6%, there was poor agreement between individual values. In both measurements, the 'V' volume of urea distribution was calculated using Watson's equation. Studies consistently show that this equation overestimates total body water [21-23]. Several other methods for estimating the total body water volume in hemodialysis patients are suggested, including the St. Michael's Hospital (SMH) equations, which showed a good correlation [24]. Minor errors in the estimation of volume 'V' might affect the Kt/*V*_Dau_ least as it is used only for a small correction in the Daugirdas formula but can result in significant errors in the measurement of dialysis dose using OCM.

Most study subjects were presumed to have achieved dry weight, as only the patients who had been on dialysis for at least two months were enrolled. However, volume status was assessed only by clinical examination. Mild pedal edema was present during clinical examination in 11 of our patients (22%). Our study did not use objective methods like body composition monitors (BCM). When BCM was used to identify 'V', studies showed better agreement between Kt/*V*_OCM_ and Kt/*V*_Dau_ [25,26].

Discrepancy analysis

Wide Range of Dialysis Adequacy in Our Patients

The F6 HPS dialyzer was used. Since the patients had high differences in weight (V), those with lower weights had better clearance than those with higher weights and vice versa. The use of an F8 HPS dialyzer would have resulted in better overall clearances in these patients.

Variability in duration: Duration was limited for some patients who developed acute complications during dialysis, resulting in lower clearance in those patients.

Poor Correlation Between Kt/V_OCM_ and Kt/V_Dau_

Dry weight assessment: The majority of study participants were assumed to be of dry weight due to the inclusion of dialysis patients for at least two months. However, volume status was assessed only by clinical examination. Since it is used for the estimation of Kt/*V*_OCM_, errors in dry weight would affect the value.

Hematocrit: Similar to dry weight assessment, the exact hematocrit of the patient just before the measurement dialysis session could not be assessed as the patients may not wait for the reports, causing inconvenience. For practicality, the last hematocrit measured during the monthly investigations was used, whose variability also could have resulted in errors in Kt/*V*_OCM_.

Volume assessment: The Watson equation was used for volume assessment in our study. As indicated earlier, this could result in significant errors in Kt/*V*_OCM_ rather than Kt/*V*_Dau_, resulting in poor correlation.

Errors in sampling: Though samples were planned to be taken immediately after the dialysis session, either with a stop-dialysate-flow method (for 3 minutes) or a slow-flow method (reducing blood flow to 100 mL/min for 15 seconds), sampling errors could not be ruled out for all patients, resulting in variability.

OCM device: Since the Fresenius 4000S machines used were of different ages (not used for similar durations), wear and tear of the components could have resulted in variable Kt/*V*_OCM_.

The limitations of our study are to be noted. First, the measurement of the adequacy of small solute clearance during intermittent hemodialysis is most rigorously based on formal urea kinetic modeling [27]. Still, only Daugirdas' second-generation formula for single-pool Kt/V was used as a standard in our study. Second, the urea distribution volume was estimated by an anthropometry-based equation (Watson's equation), which may not represent the actual volume status of the patients. The use of BCM could have yielded relatively accurate body-water estimations. Third, some studies show an excellent correlation between equilibrated Kt/*V*_Dau_ and Kt/*V*_OCM_ [28]; this double-pool measurement of Kt/V was not carried out due to patient inconvenience. Lastly, our data included only 50 sessions of intermittent dialysis to assess the correlation between different methods of dialysis adequacy.

## Conclusions

In conclusion, our findings illustrate that using URR may be a more straightforward and reliable means for assessing the adequacy of intermittent hemodialysis with minimal errors in ESRD patients compared to Kt/*V*_OCM_. URR also correlates to patient outcomes similar to dialysis doses measured as Kt/*V*_Dau_ in multiple studies. An online clearance monitor poorly correlated measurements with the Daugirdas' second-generation formula for single-pool Kt/V, the standard recommended by NKF-KDOQI. Hence, though online clearance monitors offer easy estimation and practicality with minimal effort, they are prone to multiple errors and may not be accurate in some settings.
